# Retraction Note: Protective effects of gingerol on streptozotocin-induced sporadic Alzheimer’s disease: emphasis on inhibition of β-amyloid, COX-2, alpha-, beta - secretases and APH1a

**DOI:** 10.1038/s41598-023-36652-w

**Published:** 2023-06-09

**Authors:** Ali M. El Halawany, Nesrine S. EL Sayed, Hossam M. Abdallah, Riham Salah El Dine

**Affiliations:** 1 Department of Pharmacognosy, Faculty of Pharmacy, Cairo University, Cairo, 11562 Egypt; 2 Department of Pharmacology & Toxicology, Faculty of Pharmacy, Cairo University, Cairo, 11562 Egypt; 3 Department of Pharmacology & Toxicology, Faculty of Pharmacy & Biotechnology, German University in Cairo, Cairo, 11835 Egypt; 4 Department of Natural Products, Faculty of Pharmacy, King Abdulaziz University, Jeddah, 21589 Saudi Arabia

Retraction of: *Scientific Reports* 10.1038/s41598-017-02961-0, published online 06 June 2017

Editors have retracted this Article.

After publication of this Article it has been brought to Editors attention that several panels in Figure 8 contain repetitive features in Figure 8a and b, and between-image duplications (Figure 8d and e). The Authors were not able to provide the raw data for this figure. The Editors therefore no longer have confidence in the reliability of the data presented.

Hossam M. Abdallah disagrees with the retraction. Ali M. El Halawany, Nesrine S. EL Sayed, & Riham Salah El Dine did not respond to the correspondence from the Editors about this retraction.

